# Eomesodermin of Atlantic Salmon: An Important Regulator of Cytolytic Gene and Interferon Gamma Expression in Spleen Lymphocytes

**DOI:** 10.1371/journal.pone.0055893

**Published:** 2013-02-07

**Authors:** Jaya Kumari, Jarl Bøgwald, Roy A. Dalmo

**Affiliations:** Norwegian College of Fishery Science, Faculty of Biosciences, Fisheries and Economics University of Tromsø, Tromsø, Norway; University of California, Riverside, United States of America

## Abstract

Eomesodermin (Eomes), a T-bet homologue expressed in activated CD8+T cells was recently proposed to act as a master regulator of cytotoxic CD8+ T cell effector function and offers an exciting avenue for future exploration. Here, we have identified and characterized the full-length Atlantic salmon Eomes cDNA (2477 bp). Promoter analysis of the salmon Eomes showed the presence of important putative transcription binding sites like SP1, FOXO, Oct-1, SMAD, STAT, IRF, and Ets-1. The basal core region responsible for the promoter activity was located between base −199 and +59. Quantitative PCR analysis revealed that the Atlantic salmon Eomes was ubiquitously expressed in all the tissues studied but strongly expressed in the ovary, spleen, brain, and the head kidney. Moreover, the involvement of Eomes in Atlantic salmon immune response and its relation with the cytolytic activity was demonstrated by investigating the early time dependent expression profile of Eomes and CD8α followed by high interferon gamma (IFN-γ) and granzyme A expression during challenge with live *Aeromonas salmonicida and* Infectious Pancreatic Necrosis (IPN) virus. Therefore, we further analyzed the regulated expression and function of this transcription factor in spleen lymphocytes. Overexpression of Eomes induced IFN-γ, and granzyme A expression but not perforin expression, whereas small interfering RNA (siRNA) mediated suppression of Eomes expression led to significantly reduced IFN-γ production. Thus, Eomes may be critical in cytolytic gene expression and function in fish similar to mammals. Furthermore, IFN-α, and mitogens induced Eomes expression. Taken together, this is the first study on the promoter activity and regulatory role of Eomes in fish.

## Introduction

Transcription factors can have a marked effect on the fate of a cell by initiation of the gene expression patterns that determine cellular function. Therefore, a great deal of effort has been invested in identifying and understanding the individual transcription factors that influence key activities.

T-box genes belong to a highly conserved gene family that share a sequence specific DNA-binding domain of approximately 200 amino acids, called the T-box, that was first identified in the mouse *brachyury* or *T* gene [1] and suggested these genes as putative transcription factors which are important regulators of several early developmental processes [2]. Eomesodermin (Eomes) has been shown to play a key regulatory role in initiating mesoderm cell fate in most vertebrates [3] and in trophoblast differentiation in mammals [4]. Important roles of Eomes during early development have also been reported in zebrafish [5]–[7]. Two T-box genes are expressed in cells of the immune system: Eomesodermin [3] and Tbx-21 or T-bet [8].

Recent studies, however, showed that mammalian Eomes is likely to complement the actions of T-bet and act as a key regulatory gene in the development of cell mediated immunity [3]. The Eomes gene is similar to the T-bet gene and is expressed in activated CD8+ T cells, activated CD300a+ CD4 T cells [9] as well as in resting and activated NK cells [10], and also in activated γδT-cells [11]. Eomes has been shown to be involved in the effector differentiation and activation of CD8+ T cells and cytolytic functions [3], [12], plays a critical role in protective effector function of γδT-cells and offers an exciting avenue for future exploration in tumor immunity. Furthermore, recent studies have also established T-bet and Eomes as key regulators of adaptive cell-mediated immunity against cancer [13]. Overexpression and antagonism studies using dominant negative factors have suggested that Eomes and T-bet might have cooperative or redundant functions in regulating the genes encoding IFN-γ and cytolytic molecules in CD8+ T cells [3]. In addition, overexpression of Eomes has been shown to drive the expression of IFN-γ, perforin and granzyme B, thereby indicating that these genes are also direct downstream targets of Eomes [14]. These data suggests that Eomes has a pivotal role in controlling cytolytic activity of murine and human CD8 T lymphocytes and also in human γδT-cells.

In lower vertebrates, only zebrafish Eomes has been characterized and was found to be involved in zebrafish immune response. Whether this transcription factor in lower vertebrates, like teleosts possesses similar immune function in cell-mediated responses, as in mammals, is not yet known [15]. Towards delineating its function, the Atlantic salmon (genomic) *Eomes* was characterized in terms of sequence and promoter analysis, tissue expression patterns and immune response following challenge with the well-known fish pathogens *A. salmonicida* and IPN virus.

Although it has been established that Eomes plays a key role in the regulation of cytolytic activity of human and murine CD8+ T lymphocytes, its function in immune system of lower vertebrates remains to be elucidated. Therefore, based on the observations made in the murine and human system, we hypothesize that eomesodermin is present and is involved in Atlantic salmon immune response - particularly to lymphocyte function. Accordingly, we also aimed to identify a bundle of the mediators that control the expression of Eomes in fish lymphocytes using quantitative PCR.

## Materials and Methods

### Animals

Atlantic salmon weighing 70–100 g were kept at the Aquaculture Research Station (Tromsø, Norway) in circular 200 L tanks supplied with re-circulating freshwater at an ambient temperature of approximately 10°C with 12/12 h illumination, and fed a commercial pellet diet. Prior to treatment or challenge, fish were anaesthetised in 0.005% benzocaine. Fish were sacrificed using 0.01% benzocaine prior to collection of different tissues. The challenged fish were also kept at the same conditions as before challenge. The experimental protocols used for Atlantic salmon in this study were reviewed and approved by the Committee on the Ethics and Animal Welfare of Norway, the National Animal Research Authority (NARA)/(Norwegian: Forsøksdyrutvalget, FDU), http://www.fdu.no.

### Molecular Cloning and Sequencing of Atlantic Salmon (As) Eomes cDNA

A partial salmon EST sequence similar to vertebrate eomesodermin was identified based on nucleotide and amino acid sequence homology to zebrafish Eomes1 sequence in GenBank using the BLAST programme (http://www.ncbi.nlm.nih.gov/BLAST). Internal primers were designed from the AsEST sequence and AsEomes clones were obtained from the cDNA library obtained from the stimulated spleen tissue and sequenced. 3′and 5′ RACE were performed using a GeneRacer™ Kit (Invitrogen, Carlsbad, CA, USA) according to manufacturer’s instruction. Total RNA (3 µg) isolated from Atlantic salmon (∼80 g) spleen tissue using TRIZOL® Reagent (Invitrogen,) was used as a template and reverse transcribed using Superscript™ III RT and the GeneRacer Oligo dT primer. Primers used for the 5′ or 3′ RACE are listed in Table I. PCR products were gel purified using MinElute Gel Extraction kit (QIAgen, Hilden, Germany) and cloned in a TOPO vector (Invitrogen). Plasmid DNA from at least 10 independent clones was purified using QIAprep Spin Miniprep kit and sequenced. The cDNA sequence and deduced amino acid sequence of Atlantic salmon Eomes were analyzed using the BLAST program, the ExPASy Molecular Biology server (http://us.expasy.org) and Pfam [16]. Amino acid identity and similarity were done with the Matrix Global Alignment Tool (MatGAT) program v 2.0 [17] using default parameters. Multiple amino acid sequence alignments were constructed with ClustalW2 program and further edited using GeneDoc, version 2.7. Phylogenetic tree was constructed using the neighbour-joining method using the MEGA v 4.0 program [18]. The topological stability of the tree was evaluated by 10 000 bootstrap re-samplings.

### Isolation of 5′-flanking Region of the AsEomes Gene by Genome Walking

The 5′-flanking region of the Eomes gene was isolated using the Universal GenomeWalker Kit (Clontech, Palo, Alto, CA, USA). Four GenomeWalker libraries were constructed according to the manufacturer’s instructions. For each genome walker experiment two adjacent reverse primers were designed in the 5′ UTR region of the target gene (Table 1), and used in two PCRs in combination with the forward adaptor primers AP1 and AP2 (Clontech) for each library. The resulting PCR products from four different libraries were cloned in TOPO vector (Invitrogen), sequenced and analyzed as described above.

**Table 1:** List of primers and their designated applications.

Primer name	Sequence 5′-3′	Application
AsEomes-F1	CCTGTGGCTCAAGTTTCACCGATTC	partial cDNA cloning
AsEomes-F2	CCGACAGCCTCCCCTCGCTCTCA	3′ RACE
AsEomes-NF	CAGCCCTTCTTCCAGGACCAGTTTG	3′ RACE
AsEomes-R1	GGGTGACAAACCAGCGCTGAG	partial cDNA cloning
AsEomes-R2	CGGCCAACACGACCTCAACAAATAC	5′ RACE
AsEomes-NR	CCGCCTGCCCTGTTTAGTGATGATC	5′ RACE
AsEomes-PriR	GGCTACAACCATCAAGACGCTACACCACTG	Genome walking
AsEomes-SecR	CTAATGTTGACCTTCTCAGTTGCACCTAC	Genome walking
AP1	GTAATACGACTCACTATAGGGC	Genome walking
AP2	ACTATAGGGCACGCGTGGT	Genome walking
AsEomesF3	CCCAATTACTTTGACATGTTACGGGAAG	Promoter check
AsEomesR3	GCTATTATTTGCACTATCTGACGACGAGAG	Promoter check
AsEomesMluIF1	CG A CGC GT ATG GTT AGC CAA ATG TCA ATT ATG G	SEAP construct
AsEomesMluIF2	CG A CGC GT ATC TCC CAA TTA CTT TGA CAT GTT ACG	SEAP construct
AsEomesMluIF3	CGACGCGTGACAGGCTCATTTTCCCATTTAACTC	SEAP construct
AsEomesBglIIR	GAAGATCTGACGCTACACCACTGCGCCCTGGTATG	SEAP construct
AsEomesSalIF1	ACGCGTCGACATG GTT AGC CAA ATG TCA ATT ATG G	pMet Luciferase construct
AsEomesSalIF2	ACGCGTCGACATC TCC CAA TTA CTT TGA CAT GTT ACG	pMet Luciferase construct
AsEomesSalIF3	ACGCGTCGACCGACAGGCTCATTTTCCCATTTAACTC	pMet Luciferase construct
AsEomesSacIIR	TCCCCGCGGGCTACACCACTGCGCCCTGGTATG	pMet Luciferase construct
M13 F	CAGGAAACAGCTATGAC	Sequencing
M13 R	GTAAAACGACGGCCAG	Sequencing
T3 F	ATTAACCCTCACTAAAGGGA	Sequencing
T7 R	TAATACGACTCACTATAGGG	Sequencing
pMetLuc-R	CACGATGTCGATGTTGGGG	Sequencing
pSEAP2-F	CTAGCAAAATAGGCTGTCCC	Sequencing
pSEAP2-R	CCTCGGCTGCCTCGCGGTTCC	Sequencing
AsEomes-F	ACCTCTCGTCGTCAGATAGTG	Real time PCR
AsEomes-R	GGACCGGTGAGTCTTTTCTTC	Real time PCR
AsPerforin 1-F	GTGTGTCCTGCGGGTATTAC	Real time PCR
AsPerforin 1-R	CCTGGCACTGCATGATACTG	Real time PCR
AsIFN-γ-F	CCACTATAAGATCTCCAAGGAC	Real time PCR
AsIFN-γ-R	CTCCTGAACCTTCCCCTTGAC	Real time PCR
AsGrzA-F	GGTGTTTCTAGGGGTCCACTC	Real time PCR
AsGrzA-R	TGCCACAGGGACAGGTAACG	Real time PCR
AsT-21-F	GGCATAGGTGGCAATCTTTACC	Real time PCR
AsT-21-R	GTGCCGATCCGCCCTGTC	Real time PCR
AsEFα-F	CGGCAAGTCCACCACCAC	Real time PCR
AsEFα-R	GTAGTACCTGCCAGTCTCAAAC	Real time PCR
AsEomes/myc/XhoI-F	CCG CTC GAG CGG ATG GAA CAA AAA CTC ATC TCA GAA GAG GAT CTG ATG CAG CTG GAG AAT ATT CTT C	Myc tagged expression construct
AsEomes/EcoRI-R	CCG GAA TTC CGT TAG GGG CTT GCG TAA AAG GCA TAG	Expression construct

Note: Restriction endonuclease site are underlined.

In order to verify this new sequence, a forward primer (AsEomes-F3) was designed within this new sequence and used with a reverse primer (AsEomes-R3) designed within the transcribed region of the Eomes gene (Table 1). PCR from the Atlantic salmon genomic DNA was performed and the products obtained were cloned and sequenced. Identification of transcription factor binding motives was performed with TRANSFAC® (Biobase International) and MatInspector version 6.2 [19].

### Construction of Reporter Gene Plasmids

Deletion constructs with successive removal of the 5′ region were generated by PCR using the forward primers AP1, AsEomesMluIF3, AsEomesMluIF2, and AsEomesMluIF1 having recognition sequences for restriction endonuclease MluI while the reverse primer (corresponds to region 59 bases downstream to TSS) AsEomesBglIIR (Table 1) had an BglII restriction site to generate the constructs p(2003/+58), p(1061/+58), p(−518/+58), and p(−199/+58), respectively. Similarly, the same 5′ deletion construct was made in parallel using forward primers having SalI and reverse with SacII restriction sites (Table I). The promoterless pMet Luciferase Reporter and pSEAP2-Basic Vectors were used as reporter plasmids for cloning. Both the PCR products of different 5′ deletion constructs and the basic reporter vectors were digested with their respective restriction enzymes (New England BioLab Inc., Ipswich, MA, USA) and ligated (T4 DNA ligase) to generate the above mentioned constructs for each basic reporter vector (pMet Luciferase and pSEAP2) in parallel. All plasmid DNA constructs were isolated using Plasmid Mini Kit (Qiagen) to have pure quality plasmid for transfection. All plasmid constructs were verified by restriction map analysis and by DNA sequencing.

### Cell Culture, Transfection and Reporter Activity Assay

HeLa cells (ATCC CCL-2) were grown as described elsewhere [20]. The day before transfection, 2×10^4^ cells were seeded per well of a 96 well plate (Nunc) in 100 µl growth medium. The cells were co-transfected with 0.3 µg of the different Eomes promoter-pMetLuc reporter constructs, promoterless pMetLuc Reporter, and pMetLuc control vector and 0.1 µg of different Eomes promoter-SEAP Reporter constructs, promoterless pSEAP basic, and pSEAP2-Control vector (for normalizing transfection efficiency) using Polyfect Transfection Reagent (QIAgen) according to the manufacturer’s instructions. The plasmid DNA of each construct was prepared using the Plasmid Mini Kit (QIAgen). 24 h after transfection, the media were removed and replaced by media with or without 100 ng/ml *A. salmonicida* LPS (gift from Tim Bowden and Ian Bricknell, FRS Marine Laboratory, Scotland) to activate the signal transduction pathway. After 24 h of incubation with LPS, the culture medium was collected and analyzed for Metridia luciferase using Ready-To-Glow™ Secreted Luciferase Reporter System (Clontech) and SEAP activity using Great EscAPe™ SEAP Chemiluminescence Detection Kit (Clontech). Then the luciferase and SEAP activity were assayed using a plate Luminometer, Luminoskan Ascent (Thermo Electron Corporation, Finland). The experiment was carried out in triplicate for each construct, and the Luciferase activity was normalized to the SEAP activity.

### Tissue Specific Expression of AsEomes

The expression pattern of Eomes in different tissues was measured by real-time PCR. Total RNA from the tissues of liver spleen, head kidney, gill, thymus, intestine, heart, skin, brain, ovary and testis was extracted from Atlantic salmon by the TRIZOL® method.

### *In vivo* Infection

#### Bacterial infection

Atlantic salmon (80–100 g) were injected intraperitoneally (i.p) with a 100 µl suspension of 1.3×10^6^ colony forming units of live *Aeromonas salmonicida* (virulent strain, LFI 4017, originally isolated from Atlantic salmon infected with furunculosis) in PBS and the fish controls were injected with PBS. Sampling of head kidney and spleen was performed at 0 h, 4 h, 24 h, 48 h, and 96 h post injection from six fish per group.

#### Viral infection

Atlantic salmon parr were challenged in a cohabitation model by adding 12% of IPN virus shedders to the total biomass in each tank. The virus shedders were intraperitoneally injected with 1×10^7^ TCID_50_/fish of a highly virulent IPNV Norwegian SP strain NVI015 (GenBank accession no. **AY379740**). At 4, 7, 14, 21 and 56 days post challenge head kidney and spleen samples were collected from 6 fish at each time point.

All organs were rapidly transferred to and kept in RNA-later (Ambion, Austin, TX, USA) and subsequently processed for RNA isolation. Total RNA was extracted by TRIZOL® (Invitrogen) according to manufacturer’s instruction. To remove any contaminating genomic DNA, samples were treated with DNase (TURBO DNA-free™, Ambion). Quantitative real-time PCR analysis was performed as described below.

### Primary Cell Culture and Stimulation

Three fish (1–1.5 kg) were anaesthetized with benzocaine, the spleen was aseptically removed and placed in universal tubes containing Leibowitz medium (L-15) containing 100 U/ml penicillin, 100 µg/ml streptomycin, 0.5% FBS and 40 U/ml heparin (referred herein as L-15i). The tissue was gently pushed through sterile 100-µm mesh screens and the screens were rinsed with L-15i. The resulting cell suspension was layered on 25/54% Percoll gradient (GE Healthcare, Nydalen, Oslo, Norway) and centrifuged at 400×g for 20 min at 4°C. Cells at the 25/54% Percoll interface were collected, washed twice in L-15i by centrifugation at 400×g for 10 min at 4°C, and the leukocytes were then re-suspended in complete medium (the same as incomplete medium but with 5% FBS) at 2×10^6^ cells/ml. One millilitre of cells were seeded in 12 well tissue culture plates and incubated at 14°C for 24, 48, and 72 h for each stimulant or combination of stimulants. The leukocytes were stimulated with known mitogens for T-cell stimulation i.e. PHA (10 µg/ml)+ConA (10 µg/ml)+human IL-2 (1 ng/ml), and with recombinant Atlantic salmon IFN-α2 at two dose levels: 5 ng/ml and 0.5 µg/ml.

### Cloning of Eomesodermin cDNA and Transient Transfection of Primary Spleen Lymphocytes

The N-terminal myc tagged full length AsEomes cDNA was cloned into the XhoI and EcoRI site of the pcDNA3.1 expression vector (Invitrogen).

Spleen leukocytes were transfected with 3′modified Alexafluor 647Eomes-siRNA (small interfering RNA) and contol siRNA (Qiagen, Hilden, Germany), Eomes/pcDNA3.1 vector or pmaxGFP vector (Amaxa, Cologne, Germany), using the Human T Cell Nucleofector Kit (Amaxa). After 4–5 h of nucleofection, the leukocytes were stimulated with PHA (10 µg/ml)+ConA (10 µg/ml)+human IL-2 (1 ng/ml) for 24 hours at 14°C. After transfection, cells were analyzed by flow cytometry.

### Flow Cytometry and RT-PCR of Sorted Cells

Following treatment of spleen leukocytes at different time intervals, propidium iodide, (PI) (50 µg/ml) was added to each 1 ml cell suspension to stain dead cells. Fluorescence-activated cell sorting was performed based on PI exclusion, forward scatter (FS), and side scatter (SS) using BD FACSAria™ Flow Cytometer (BD Bioscience). Spleen lymphocytes were sorted based on their size. Total RNA was isolated from 10,000 spleen lymphocytes using the RNeasy Micro Kit (Qiagen, including DNase digestion). Purified RNA was confirmed to be intact by gel electrophoresis while RNA concentration and purity were measured spectrophotometrically (Nano-Drop Technologies, Wilmington, USA). The synthesis of cDNA was performed with Taqman RT Reagents (Applied Biosystems, CA, USA) using random hexamers and 500 ng total RNA as template in a 50 µl reaction volume.

### Analysis of Gene Expression by Real-time PCR (QPCR)

QPCR was performed in duplicates with an ABI PRISM 7500 Fast Real-Time PCR System (Applied Biosystem) using Fast SYBR® Green (Applied Biosystem). Reaction mixtures were incubated for 10 min at 95°C, followed by 40 cycles of 15s at 95°C, 1 min at 60°C, and finally 15s at 95°C, 1 min 60°C, and 15 s at 95°C. For each mRNA, gene expression was normalized by the EF-1α in each sample. The primers used are shown in Table1.

### Immunofluorescence Analysis

Atlantic salmon spleen primary lymphocyte cells transfected with siEomes and myc pcDNA3.1/Eomes plasmid were incubated on poly-L-lysine coated coverslip and fixed with 4% paraformaldehyde. Coverslips were incubated sequentially with 0.2% Triton X-100 (Sigma), blocking buffer (PBS with 5% FBS), primary antibodies [anti-zebrafish Eomes pAbs (1∶1000 dilution) and anti-myc monoclonal antibody (1∶500 dilution) Covance, Princeton, New Jersey] in blocking buffer, and fluorescent secondary antibodies, Alexa Fluor 546 and 594 (Invitrogen) were added at a 1∶1000 dilution in the blocking buffer. The cells were again washed three times with PBS and counter-stained with DAPI (2 µg/ml) (Sigma), then mounted using anti-fading mounting medium (DABCO). Confocal microscopy was performed using a Leica TCS SP5 confocal microscope (Leica Microsystems Gmbh, Mannheim, Germany) equipped with a 63X1.2 NA water immersion objective. The excitation wavelengths for DAPI, Alexa Fluor 546, and Alexa Fluor 594 were 405 nm, 561 nm, and 594 nm, respectively. The emission band passes were 450–500 nm, 565–615 nm, and 600–630 nm, respectively. Live images for studying positive transfection with si-Eomes RNA were acquired using an LSM510 META laser scanning confocal microscope (Carl Zeiss GmbH, Jena, Germany) equipped with a 40X 1.2 NA water immersion objective. Alexa Fluor 647 was excited using a 633 nm HeNe laser, and the emission band pass was 644–719 nm.

### Statistical Analysis

Data were analyzed by one-way analysis of variance (ANOVA) followed by Tukey test using SPSS 19.0 software. Differences were considered statistically significant when *P*<0.05.

## Results

### Sequencing and Characterization of Salmon Eomesodermin

**Figure 1:**
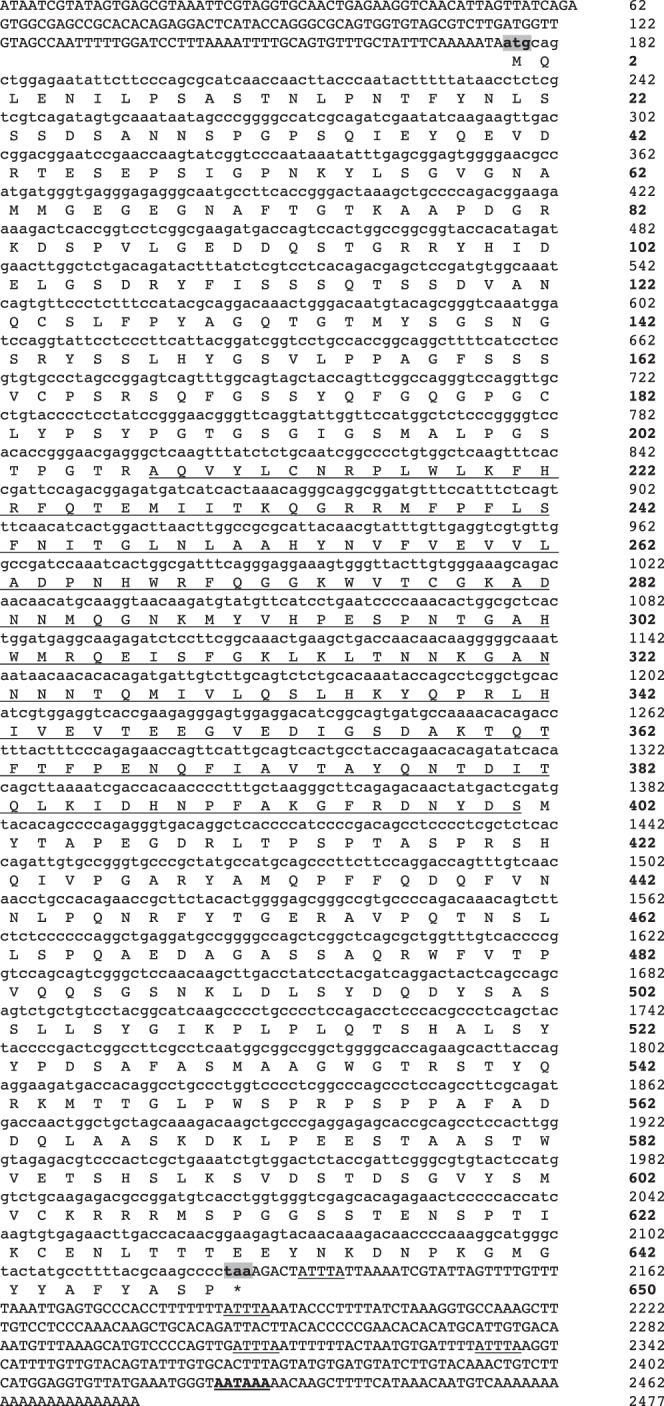
Nucleotide and deduced amino acid sequence of Atlantic salmon Eomes cDNA. Uppercase denotes the UTR’s and lowercase denotes the coding regions. The T-box domain is underlined. Start and stop codons are shaded and marked with bold letters. The asterisk indicates the stop codon. The RNA instability motif (ATTTA) is underlined. The putative polyadenylation signal is bold and underlined.

We identified the full-length cDNA sequence of Eomes (GenBank accession number **EU418014**) from Atlantic salmon by 5′ and 3′ RACE. The Atlantic salmon Eomes cDNA consisted of 2477 bp in length including a 176 bp 5′ untranslated region (UTR), a 348 bp 3′ UTR and possessed an open reading frame of 1953 bp. Four mRNA instability motifs (ATTTA) and one polyadenylation signal (ATTAAA) were found followed by a poly (A) tail in the 3′ UTR. The putative Eomes protein in Atlantic salmon was predicted to be 650 aa with a molecular weight of 71 kDa and pI of 6.37. The conserved T-box DNA binding domain consisted of 194 aa (Figure1).

Figure S1 shows the predicted aa sequences of salmon Eomes and in other vertebrates. Salmon Eomes shared 79.3% and 58.2% amino acid identity with zebrafish Eomes1 and human Eomes, respectively (Table S1). Phylogenetic analysis showed that salmon Eomes claded with zebrafish Eomes1 and was much closer to frog and mammalian Eomes than to T-brain1 and T-bet of other vertebrate species (Figure S2).

### Structure of 5′ Flanking Region of AsEomes Gene

**Figure 2:**
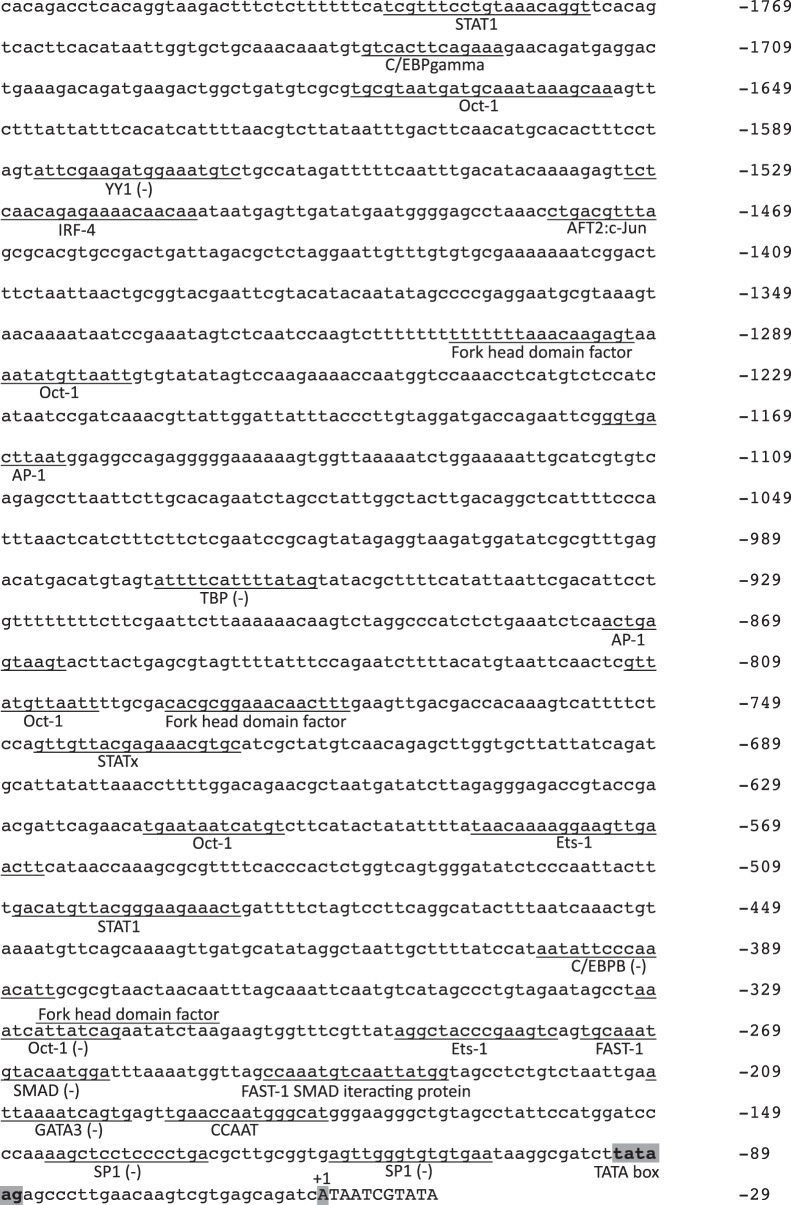
Atlantic salmon Eomes promoter sequence **with consensus transcription factor binding sites.** The consensus transcription binding sites were predicted by MatInspector and TRANSFAC. Putative transcription factor binding sites are underlined, while (−) sign indicates the binding sites identified on the negative strand. TATA box and transcription start site (+1) are shaded.

As the first step towards understanding the transcriptional regulation of the salmon Eomes gene, a sequence of 1,949 bp lying 5′ to the TSS was determined (GenBank accession no. **FJ360615**). Sequence analysis using the transcription factor binding site prediction program MatInspector and TRANSFAC revealed several notable features. Promoter analysis of the salmon Eomes revealed the presence of important putative transcription binding sites like SP1, Fork head domain factor (FOXO), Oct-1, SMAD, STAT, Ets-1, FAST-1 SMAD interacting protein, and IRF 4 and 7 on the 5′ flanking region of the salmon *Eomes* gene (Figure 2)**.** Furthermore, in the 5′ flanking region of AsEomes, TATA box was observed in the region close to the transcription initiation site i.e. between nt −33 and −27 position upstream of transcription start site (TSS). Lastly, CCAAT box was present at position nt −127 which is also known to be important for transcriptional regulation of genes in T cells. Other putative transcription factor-binding sites are shown in Figure 2.

### Functional Mapping of AsEomes Promoter

To analyse the promoter activity of the obtained 5′ flanking region of the AsEomes gene, the promoter-reporter plasmid p(−2003/+59) construct was transfected into the HeLa cell line and the putative promoter driven luciferase activity was measured. A 45-fold increase of luciferase activity (normalized to SEAP activity) compared to promoter-less controls (pMetLuc-Reporter) was obtained, indicating that the cloned 5′-flanking region represented the functional promoter of the AsEomes gene.

**Figure 3:**
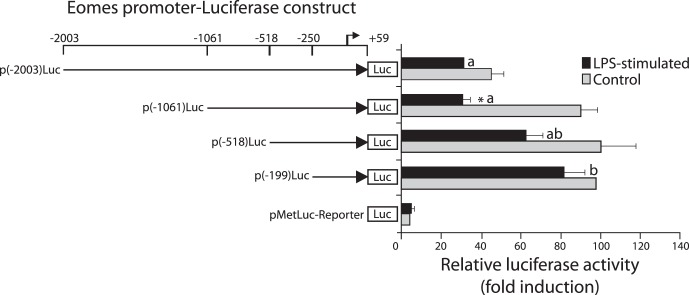
Structural and functional analysis of the 5′-upstream region of the salmon Eomes gene. HeLa cells were transiently transfected with 0.3 µg of Eomes promoter/luciferase plasmid and 0.1 µg of Eomes promoter/SEAP plasmid (internal control). HeLa cells were stimulated with LPS and after 24 h luciferase activities were measured. Units of luciferase activity were normalized to activity of cotransfected pSEAP (relative luciferase activity). The error bars represent S.E.M values (n = 3). Asterisk (*) above the bars shows significant difference (*P*<0.05) compared to respective control and the different letters (a and b) on the bars denote significant difference compared to the smallest construct. The bent arrow represents the salmon Eomes transcriptional start site.

To precisely define the 5′ end of TSS of salmon Eomes, progressive deletion constructs of the AsEomes promoter region were generated and transiently transfected into HeLa cells, commonly used as host cells for transfection experiments, and were subsequently stimulated with *A. salmonicida* LPS. These results suggested that a basal core region responsible for the promoter activity was located between nucleotide −199 and 59, which contained the TATA and CCAAT box. Reporter gene activity observed in construct p(−518) showed no significant decrease when the promoter sequence was extended to include an additional 199 bp upstream when treated with LPS. Longer promoter fragments p(−1061 and −2003) exhibited a significant decline of 50–60% in transcriptional activity compared to the basal promoter construct p(−199) (*P*<0.05). This showed that the LPS suppressive promoter activity was chiefly dependent on the cis-elements located in the DNA region between −1061 and −2003 bp of the deletion construct (Figure 3). Thus, the pattern suggests the presence of a negative regulatory element between −518 and −1061 bp in the AsEomes promoter caused by LPS stimulation.

### Tissue Distribution of AsEomes Expression in Healthy Salmon

**Figure 4:**
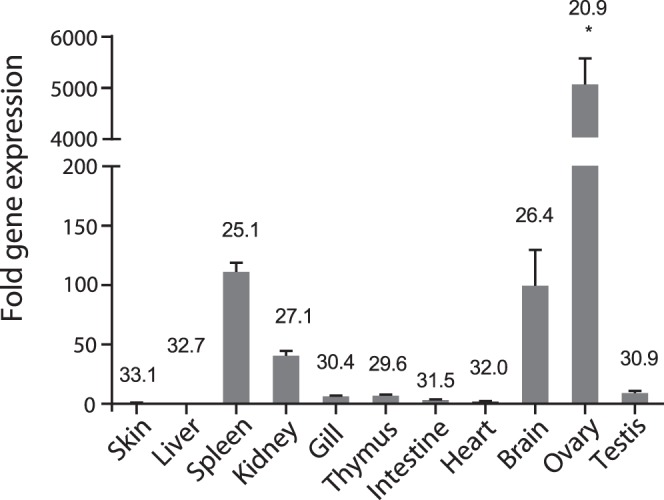
Tissue distribution of Eomes expression in Atlantic salmon. Expression of salmon Eomes in different organs as detected by real-time PCR. Gene expression data were normalized to EF-1α expression using skin as a calibrator. Bar represents the mean ± S.E.M (n = 6). Asterisk (*) above the bar shows significant difference (*P*<0.05) compared with the organ that showed the lowest expression (skin). The value above the bars shows average real-time CT values of six fish.

As shown in Figure 4, AsEomes was expressed in all the tissues sampled but strongly expressed in the ovary, spleen, brain, and the head kidney, with the lowest expression level in the skin.

### *In vivo* Regulation of AsEomes Expression

To elucidate the mechanism that regulates Eomes expression *in vivo* and its modulation during bacterial and viral immune responses, we utilized challenge model based study with *A. salmonicida* and IPN virus. We first examined the mRNA expression levels of AsEomes, IFNγ, CD8α, and granzyme A at different time intervals following bacterial and viral infection in the spleen and head kidney.

**Figure 5:**
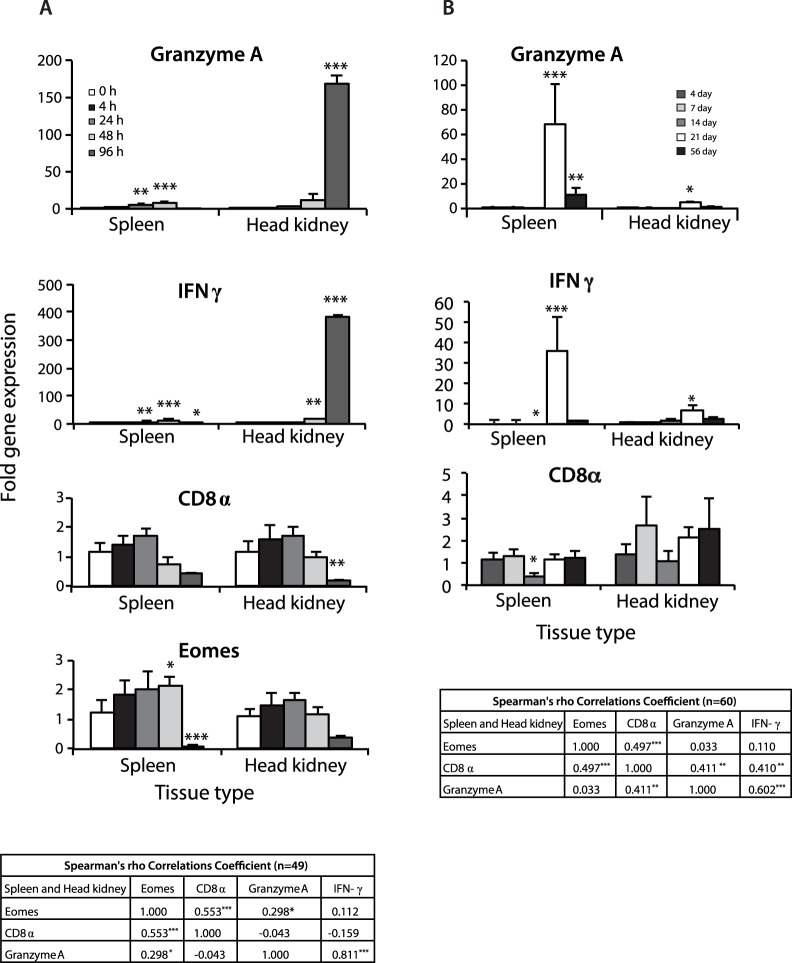
*In vivo* regulation of Eomes expression in Atlantic salmon post infection. (A) Tissue specific expression of granzyme A, IFN-γ, CD8α, and Eomes at different time-points after *A. salmonicida* challenge, and the overall correlation between them shown from top to bottom respectively, in the left panel. (B) Tissue specific expression of granzyme A, IFN-γ, CD8α, at different time-points after IPN virus challenge, and the overall correlation between them including Eomes shown from top to bottom respectively, in the right panel (Eomes expression not shown since there was no significant differences). Data were normalized to EF-1α expression at each time-points and presented as mean ± S.E.M (n = 6). Statistical differences (*P*<0.05, *P*<0.01, and *P*<0.001) between different time-points compared to control are indicated by asterisk (*, **, and ***) respectively, above the bars.

Following *A. salmonicida* infection, spleen AsEomes transcript level was significantly higher at 48 h, and the level significantly dropped on day 96 h post challenge. In the head kidney, there was a slight increase till 24 h post challenge before the expression decreased to a “resting” level at 48 h. CD8α expression in both spleen and head kidney followed a similar time-dependent expression pattern with an increase to 24 h followed by a decrease at 96 h post challenge. The expression of the key genes for cell mediated responses such as a granzyme A (CTL response) and IFNγ were significantly higher at 24 and 48 h post challenge compared to controls, followed by a decreased expression at 96 h in the spleen and in head kidney. The expressions in the head kidney were delayed compared to the spleen. Statistical analysis showed an overall strong correlation of Eomes expression with CD8α and granzyme A, as well as between granzyme A and IFN-γ expressions post infection (Figure 5A).

During the cohabitation challenge experiment with IPNV, the virus shedders started to die on day 6 post-challenge, whereas the cohabitants started dying on day 19 post challenge. The gene expression of granzyme A and IFNγ increased significantly at 21 days post IPNV challenge. CD8α and Eomes showed similar pattern of expression without any significant increase. The expression of CD8α showed a strong correlation with Eomes, IFNγ and granzyme A, suggesting that they were interacting (Figure 5B).

### *In vitro* Regulated Expression in Spleen Lymphocytes

**Figure 6:**
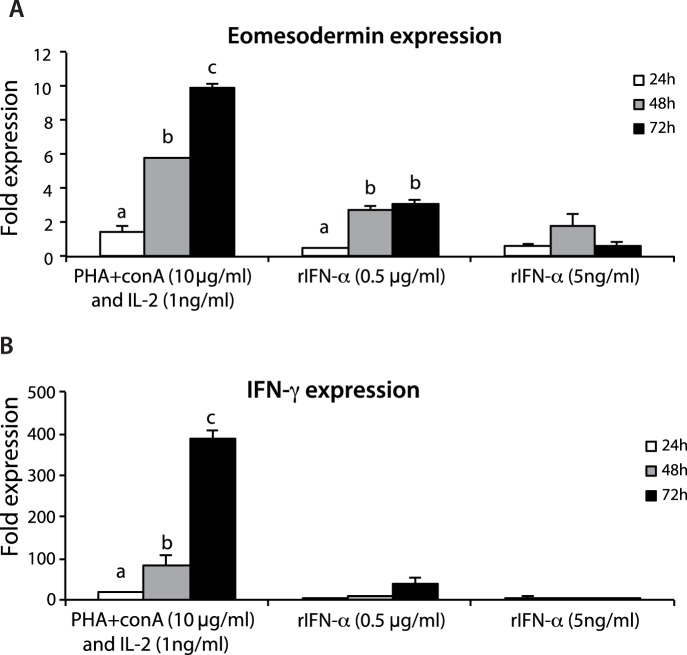
Regulators of Eomes expression in salmon lymphocytes. Spleen leukocytes were stimulated for 24 h, 48 h, and 72 h with ConA+PHA+huIL-2, IFN-α (0.5 µg/ml, and 5 ng/ml), and the mRNA levels of Eomes (A), and IFN-γ (B) were determined by real-time PCR. Gene expression is normalized against EF-1α and is shown relative to the mean of the non-stimulated cells. Each bar represents the mean ± SE of triplicate samples. Different letters denote statistically significant differences between the groups.

A bundle of stimulants, some of them known to activate lymphocytes, were tested for their potency to regulate the expression of Eomes and IFN-γ in spleen lymphocytes isolated by cell sorting. Interestingly, stimulation of spleen lymphocytes with known T cell stimulants i.e. PHA+ConA and recombinant huIL-2 resulted in the strongest and most significant induction of Eomes expression upon 72 hours of incubation. Stimulation with recombinant salmon IFN-α2 (0.5 µg/ml after 48 and 72 hours) also induced significant higher expression of Eomes compared to control cells (Figure 6A). Similar to expression of Eomes mRNA, the IFN-γ expression was also significantly increased after stimulation with the mitogens, whereas rIFN-α2 (0.5 µg/ml) induced IFN-γ expression though not statistically significant (Figure 6B).

**Figure 7:**
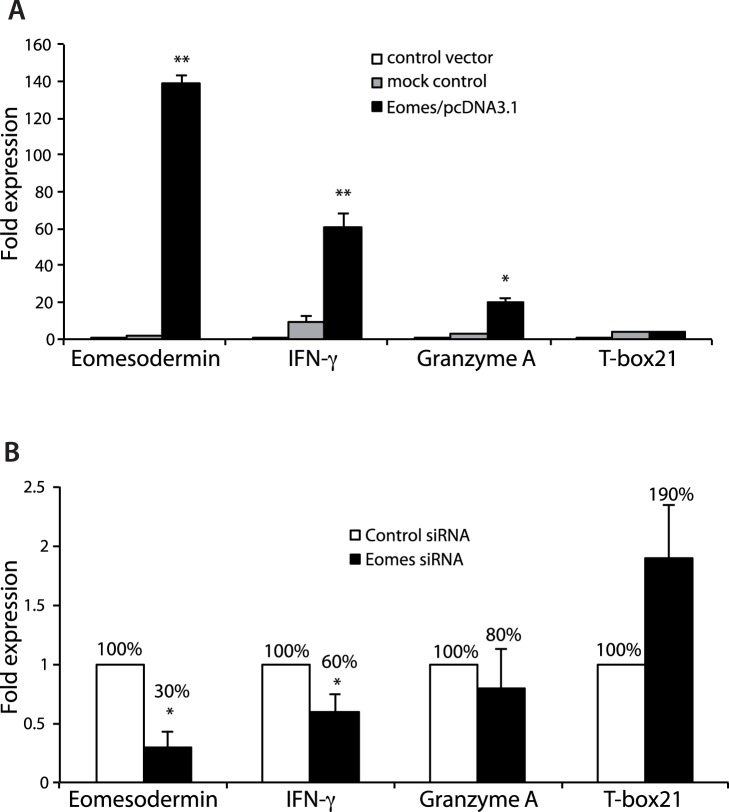
Eomes mediated effects on salmon spleen lymphocytes. Salmon leukocytes were transiently transfected with Eomes/pcDNA3.1 vector or control vector (pmaxGFP vector) (**A**) EomessiRNA or control siRNA (**B**). After 5 h, cells were stimulated with ConA+PHA+recombinant huIL-2. 24 h post stimulation, lymphocytes were sorted by FACS, collected and analyzed for the expression of Eomes, IFN-γ, granzymeA, T-box21 and EF-1α by QPCR. Eomes, IFN-γ, granzymeA, T-box21 mRNA expression in cells transfected with control siRNA were set to 100%. Statistical differences (*P*<0.005, and *P*<0.0004) between different treatment and control are indicated by asterisks (*, **) above the bars, respectively.

To investigate Eomes-mediated induction on known CD8+ T cell molecules, salmon lymphocytes were transfected with Eomes cDNA expression vector. Transfection efficiency was found to be consistently greater than 50%. While over-expression of Eomes up-regulated the expression of IFN-γ and granzyme A (Figure 7A), the expression of perforin remained undetectable in both Eomes expressing cells and under silenced conditions (data not shown). Furthermore, siRNA mediated silencing of Eomes expression in the salmon lymphocytes led to abrogated expression of the cytolytic gene granzyme A as well as of IFN-γ but with slight increase in T-box 21 expression (Figure 7B). Taken together, the expression of the cytolytic gene granzyme A and the proinflammatory cytokine IFN-γ in the salmon lymphocytes were mainly controlled by Eomes. Moreover, silencing or augmenting Eomes expression did not have significant effect on T-box 21 (T-bet) expression in spleen lymphocytes.

**Figure 8:**
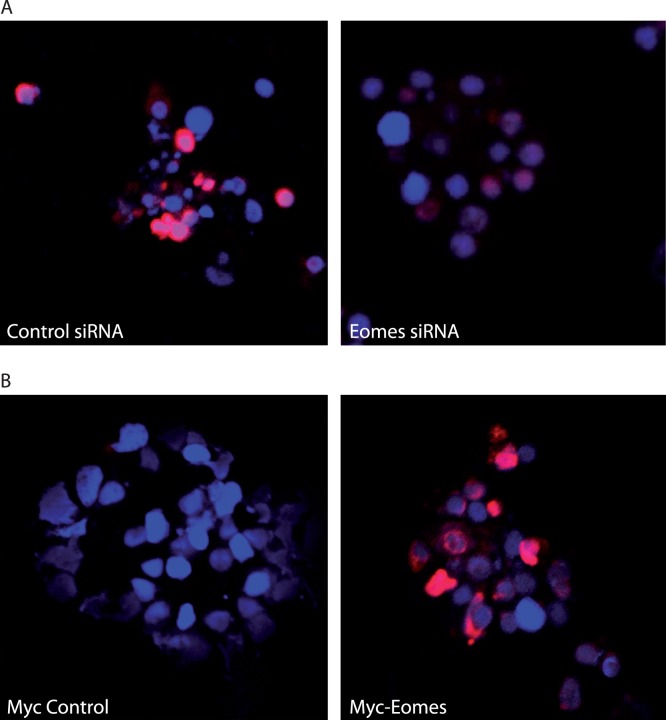
Immunofluorescence **study following** ***in vitro*** **modulation of Eomes in Atlantic salmon.** (**A**) siRNA knockdown of Eomes expression (shown in pink) in spleen lymphocytes after 48 h. (**B**) Myc-labelled Eomes ectopic expression was detected with fluorescence labelling (pink) using alexafluor 594. Nuclear staining was performed with DAPI (blue).

We further analyzed the effects of Eomes-specific siRNA on Eomes protein expression after 48 h of transfection in spleen cells using immunostaining with anti-zebrafish Eomes antibody. Immunostaining was drastically reduced by the gene-specific siRNA treatment compared to control siRNA-transfected cells showing partial knockdown (Figure 8A). Similarly, in a transient ectopic expression system using Eomes expression vector with *myc*-codon, cells were positive for myc staining and the control expression vector transfected cells were negative for myc staining (Figure 8B).

## Discussion

In this study, we have proposed the presence of Eomes in Atlantic salmon that possesses high degree of partial amino acid homology to Eomes from other animal species. Especially, the deduced amino acid sequence showed overall highest identity with zebrafish Eomes1, while there were several identical residues in all species that were aligned to AsEomes. This work presents new knowledge on the regulated expression and function of Eomes in lymphocytes of a lower vertebrate. We obtained the full-length cDNA of Eomes and the normal tissue expression was analyzed. AsEomes was strongly expressed in ovary which is in agreement with the previous report on expression of Eomes in the oocytes of zebrafish [5], [15], and is in line with the moderate expression in major lymphoid organs such as the spleen, and head kidney [15]. The expression in brain suggests its important role in neuronal differentiation [21]. Furthermore, AsEomes transcript was highly expressed after infection, which suggests its involvement in the immune system of salmon.

Sequence analysis of 5′ flanking region of AsEomes has revealed that the presence of TATA box and CCAAT boxes in the proximal promoter (−199 bp to the TSS) are in accordance with the previous study on the Xenopus Eomes promoter [21]. More recently, FOXO transcription factors were shown to bind to a promoter site in the Eomes gene and to control its expression in differentiated CD8+ T cells [22]. The presence of FOXO transcription factor binding site in the promoter of AsEomes may indicate similar underlying mechanisms of CD8 differentiation in salmon as in mammals. By PCR-aided deletion of the putative promoter region we detected a negative regulatory element between positions −518 and −1061 bp in the AsEomes promoter following LPS stimulation. Further analysis of this element indicated that they shared a number of consensus transcription factor binding sites, including AP-1 and Oct-1 sites. This could be consistent with the fact that Oct-1 and/or AP-1 function as repressors or enhancers depending on the context [23]–[25]. Moreover, the promoter region containing the two AP-1 and Oct-1 binding sites possessed the highest responsiveness to LPS induction in terms of reporter gene activity. Together, these results suggested that these two binding sites contributed to the LPS-mediated AsEomes transcription in HeLa cells. It has been argued that HeLa cells do not respond to LPS since they lack MD-2 that cooperates with TLR4 [26]. The reason why this cell line responded well to LPS in this study is not known. The LPS used in this study is of approx. 90% purity, similar to commercial preps, and may contain other cell activating substances. The functional significance of these transcription factor-binding sites awaits further investigation of the different transcriptional regulation of the AsEomes gene.

*In vivo* post challenge studies showed acute simultaneous expression of granzyme A and IFNγ in both type of infections in a tissue specific manner. These studies are consistent with the hypothesis that differentiation of effector CD8+ T cells into effector CTLs (as defined by cytotoxic activity) occurs subsequent to, rather than during, the initial TCR stimulus. A strong correlation between Eomes, CD8α, granzyme A and IFNγ shows an interaction between these genes and thus also appears to be critical for invoking the characteristics of the cytolytic effector lineage in salmon.

To delineate the mechanisms that regulate Eomes expression *in vitro*, this study identified IFN-α, a known antiviral component [27]–[29] and a known T-cell stimulator as an important inducer of Eomes expression, which may in turn regulate the differentiation of CD8+ T cells. In addition to the antiviral effects, IFN-α exerts pleiotropic cellular effects, such as inhibition of cellular proliferation, induction of apoptosis, and differentiation as well as modulation of the immune system. The latter effect includes inhibition of T-lymphocyte activation, enhancement of the cytotoxic activity of NK cells and T lymphocytes [30].

Two T-box transcription factors, Eomes and T-bet, are important for the development of effector and memory CTL [3], [10] as well as Th1 cells [31]. Studies using deletion, overexpression or dominant negative analogues of these factors have suggested that both of them are involved in the regulation of expression of IFN-γ, granzyme A and perforin [3], [32]. Consistent with these data, except for perforin expression, the results of partial knockdown, and ectopic overexpression of Eomes has been shown to drive the expression of IFNγ and granzyme A, indicating that these genes are also direct targets of AsEomes. However, overexpression of AsEomes was unable to restore the perforin expression which is in line with the study done in higher vertebrates indicating that Runx3 might be required for Eomes to function at the Prf1 gene [33]. Nevertheless, silencing of AsEomes showed increase in T-box 21 expression that may depict a T-box21 dependent compensation for Eomes for IFNγ and granzyme A expression. Therefore, the expression of the cytolytic gene granzyme A and the proinflammatory cytokine IFN-γ in fish lymphocytes was controlled by Eomes, thus representing a similar function in fish as to higher vertebrates. Further, to relate AsEomes mediated regulatory effect on cytolytic activity similar to mammals, *in vitro* cytotoxicity assay was included in our research plan, but the lack of availability of effector cell donor fish and target cells with identical classical MHC I alleles constrained standardizing the *in vitro* T cell-mediated cytotoxicity assay with MHC class I matched effector and target cells in Atlantic salmon. As such, a “Atlantic salmon-specific” CD8+ T cell mediated cytotoxicity assay was not performed. Allospecific cytotoxic cells have been described in few fish species such as ginbuna carp [34], channel catfish [35], and rainbow trout [36], [37]. Furthermore, recent studies using MHC class I different/mismatched or xenogeneic target cells showed the involvement of MHC independent innate cells (e.g. Natural killer like cells) that mediated cytotoxicity instead of cytotoxic T-cells [37]. Moreover, if bioactive recombinant salmon IFN-γ and antibodies to CD8, CD4, NK cells and resting and effector CTLs etc. have been available a more targeted study on the precise role of Eomes in salmon could have been performed.

To conclude, these data provide novel insights to better understand the control mechanisms governing the functional activity of T lymphocytes via Eomes in teleosts. Our data suggested that the function of salmon Eomes is linked to expression of IFN-γ and cytolytic molecules, and that the expression of Eomes can be modulated using IFN-α2. Our future efforts should be aimed at determining how the two T-box transcription factors T-bet and Eomes interplay in T cell differentiation and in the resulting cytolytic functions, and also to identify the key factors that induce the establishment of the a robust cytotoxic, IFN-γ secreting phenotype. This may constitute a novel approach to improve the understanding and maybe increase the control of persistent infection in teleosts. This is imperative since viral diseases are causing huge economic losses in the aquaculture industry worldwide.

## Supporting Information

Figure S1 **Multiple alignment of Eomes in Atlantic salmon with other vertebrates Eomes.** Multiple alignment of the deduced amino acid sequences of Eomes in salmon and other vertebrates by the ClustalW2 program. Residues shaded in black are completely conserved amino acids across all species aligned, and residues shaded in grey refer to 80–90% identity. Dashes indicate gaps. The T-box DNA binding domain is indicated by solid line below the alignment. The GenBank accession numbers of the Eomes sequences are as follows: zebrafish, **XP_571754**; western clawed frog, **NP_001122124**; African clawed frog, **NP_001081810**; mouse, **NP_034266**; human, **NP_005433**.
(TIF)

Figure S2 **Phylogenetic tree showing** **the relationship between salmon Eomes and T-brain1 subfamily in other vertebrates.** The phylogram was constructed with the MEGA 4.0 software using the neighbour-joining method based on an amino acid alignment (ClustalW) of the full-length protein. Numbers beside the internal branches indicate bootstrap values based on 10000 replications. The 0.05 scale indicates the genetic distance. Accession numbers for Eomes are listed in the legend of Fig. 2. The accession numbers of other T-brain1 subfamilies are as follows: Zebrafish Eomes 2, T-brain1, and Tbet (**NP_001077044, NP_001108562, and XP_001338262**), ginbuna T-bet (**BAF73805**), frog Eomes and T-brain1(**NP_001081810, NP_001072587**), Norway rat T-bet (**NP_036896**), mouse T-brain1, and T-bet (**NP_033348, and NP_062380**), human T-brain1, and T-bet (**NP_006584, and NP_037483**), Asterina T-brain1 (**BAA93701**).
(TIF)

Table S1 **Amino acid identity and similarity (%) between different vertebrate Eomes.**
(DOCX)
