# Supplementary material for: Eomesodermin of Atlantic Salmon: An Important Regulator of Cytolytic Gene and Interferon Gamma Expression in Spleen Lymphocytes
Source: PLoS One. 2013 Feb 7;8(2):e55893. doi: 10.1371/journal.pone.0055893 (PMC3567031; doi:10.1371/journal.pone.0055893)
Supplement: Table S1 — Amino acid identity and similarity (%) between different vertebrate Eomes. (DOCX) [file pone.0055893.s003.docx]

| **Similarity**  **↓** | **Identity →** | | | | | | | |
| --- | --- | --- | --- | --- | --- | --- | --- | --- |
|  |  | **1** | **2** | **3** | **4** | **5** | **6** | **7** |
|  | Salmon | - | 79.3 | 53.3 | 64.1 | 61.6 | 59.9 | 58.2 |
|  | Zebrafish Eomes 1 | 87.7 | - | 54.4 | 61.7 | 59.5 | 58.5 | 56.8 |
|  | Zebrafish Eomes 2 | 65.7 | 65.7 | - | 51.0 | 49.1 | 46.6 | 45.5 |
|  | Western clawed frog | 76.0 | 75.7 | 63.6 | - | 93.6 | 65.6 | 65.4 |
|  | African clawed frog | 73.6 | 73.3 | 60.4 | 92.1 | - | 62.7 | 62.6 |
|  | Mouse | 70.6 | 71.4 | 57.3 | 75.0 | 74.7 | - | 86.7 |
|  | Human | 71.1 | 70.8 | 56.3 | 76.8 | 74.9 | 89.4 | - |
